# Certainty-based marking in a formative assessment improves student course appreciation but not summative examination scores

**DOI:** 10.1186/s12909-019-1610-2

**Published:** 2019-05-31

**Authors:** Wiljan J. A. J. Hendriks, Nicole Bakker, Helma Pluk, Arjan de Brouwer, Bé Wieringa, Alessandra Cambi, Mirjam Zegers, Derick G. Wansink, Ron Leunissen, Peter H. M. Klaren

**Affiliations:** 1 Department of Cell Biology, Radboud Institute for Molecular Life Sciences, Radboud University Medical Center, Geert Grooteplein 26, 6525 GA Nijmegen, The Netherlands; 2 Department of Biochemistry, Radboud University Medical Center, Nijmegen, The Netherlands; 3 Department of Genetics, Radboud University Medical Center, Nijmegen, The Netherlands; 4 Radboudumc Health Academy, Radboud University Medical Center, Nijmegen, The Netherlands; 5 Department of Animal Ecology and Physiology, Institute for Water and Wetland Research, Radboud University, Nijmegen, The Netherlands

**Keywords:** Certainty-based marking, Computer-assisted learning, Confidence-based learning, Misconceptions, Self-directed learning, Student performance, Undergraduate biomedical education

## Abstract

**Background:**

Study motivation and knowledge retention benefit from regular student self-assessments. Inclusion of certainty-based learning (CBL) in computer-assisted formative tests may further enhance this by enabling students to identify whether they are uninformed or misinformed regarding the topics tested, which may trigger future study actions including instructor consultation.

**Methods:**

Using a cross-over study design involving two out of thirteen computer-assisted formative assessments (CAFAs) of a first-year cell biology course, we compared student-instructor interactions, student learning experiences and final exam scores between two (bio)medical science student cohorts who worked with different CBL-containing CAFAs.

**Results:**

A total of 389 students participated in the study. After completion 159 (41%) filled in a questionnaire on their experience with CBL during supervised CAFAs. In the control group the median duration of student-instructor interactions was 90 s (range 60–140 s), and this increased with 20 s to 110 s (range 60–150 s) in the group working with a CBL-based CAFA. The number of interactions was similar in both groups (0.22 per student per hour, regardless of CBL inclusion). Forty percent of the students expected that CBL would positively influence their study behavior, and 23% also anticipated a positive effect on examination scores. Student examination scores, however, were not affected by CBL. Almost half of the students (43%) were in favor of CBL inclusion in future computer-assisted learning modules, whereas 33% did not see merit in including CBL in CAFAs.

**Conclusions:**

Incorporation of CBL in a single formative assessment led to a slight increase in student-instructor interaction times, but had effect neither on the number of student-instructor interactions nor on exam scores. CBL inclusion positively influenced student’s appreciation of the coursework, presumably by helping students to evaluate their mastery level and identify misconceptions. A more extensive enrollment of CBL beyond an individual formative assessment, throughout a course or a curriculum, may possibly reveal positive effects on study efficacy.

**Electronic supplementary material:**

The online version of this article (10.1186/s12909-019-1610-2) contains supplementary material, which is available to authorized users.

## Background

Educational institutions strive to improve the efficacy of student learning, especially when dealing with large groups of students and limited faculty. Stimulating student’s intrinsic motivation to study is an effective means to do so, and both formative and summative testing have proven their value in this respect [1–4]. A direct effect of testing, especially when performed repeatedly and with provision of feedback [2], builds on the common notion that rehearsal supports retrieval [5]. But testing improves learning beyond that afforded by repeated study, and this indirect effect is thought to result from altering student’s study behavior towards a performance improvement [6, 7]. Indeed, provision of self-assessment tools, such as computer-based quizzes (computer-assisted formative assessments, CAFAs), contributes to the efficacy and student-appreciation of study programs [1–5, 8–10]. Our experience with the use of supervised CAFA modules in cell biology courses for first-year (bio)medical students also pointed to a downside; students may use computer modules that provide interactive learning and training content as digital textbooks rather than as formative tests. Consequently, opportunities to identify knowledge gaps and misconceptions, and to benefit from instructor support during supervised CAFA sessions, are missed. Furthermore, computer-assisted tests address only knowledge content, and individuals who answer confidently and correctly cannot be distinguished from persons who only guessed correctly. We reasoned that student self-assessment will benefit from the inclusion of certainty-based learning (CBL) in CAFAs, by stimulating student-instructor interactions and triggering other actions that enhance study performance.

In CBL, as developed over the past decades by Gardner-Medwin and colleagues [11–14] at University College London as part of the “London Agreed Protocol for Teaching” [15, 16], not only the correctness of a student’s test answer is assessed, but also the student’s confidence in the correctness of that answer is taken into account. CBL, by evaluating the knowledge per se as well as the confidence in the display of that knowledge, has been shown to improve study efficiency and knowledge retention [17–20] and appears to be well-suited to make students self-reflect on study progress and to provide them with itemized feedback [2, 21–23].

CBL is achieved by having students answering a test question first, followed by a second question asking how confident they are about the correctness of the test answer they just provided, which is scored on a three-point scale: “sure”, “partly sure” and “not sure.” Both scores are then combined, turning a binary judgment of the test answer (correct/incorrect) into a more refined measure for knowledge retention and comprehension (Additional file 1). Students can deduce whether they have mastered the subject, whether additional study efforts are required (i.e., when correct test answers are given but not with high confidence), whether they are uninformed (admittedly unknowing) or even misinformed (i.e., when incorrect answers are given with high confidence). This latter option, i.e., to identify students who are misinformed and carry misconceptions, is a unique and powerful asset of CBL. Its standard grading system indeed hands out a 6-point penalty when students entered “confident errors”, providing a strong incentive to students to take appropriate action (e.g., consult available teaching staff). Combined scoring of knowledge and certainty questions in CBL thus informs on study progress. Furthermore, it enables provision of differentiated feedback and study directives, even at the level of individual questions.

In our endeavors to aid student self-assessment and to stimulate them to take action relating to obscurities or misconceptions, we tested the potential added value of CBL in a cross-over experiment involving close to four hundred (bio)medical students during a first-year cell biology course. The number and duration of student–instructor interactions during CAFAs, students’ appreciation of formative assessment via this technique, and the ultimate summative exam results were analyzed. We conclude that the implementation of CBL in two of our CAFA modules was appreciated by students and led to a slight increase in the duration of instructor consultations but it remained without measurable effects on exam grades.

## Methods

### Subjects

During a four-week Cell Biology course at the Radboud University Medical Center (Nijmegen, The Netherlands), first-year medical (*n* = 336) and biomedical science (*n* = 126) students took thirteen subsequent computer-assisted formative assessment (CAFA) modules, usually in teams of two to three persons per computer terminal. Subjects covered in the CAFA series were: 1 - The cell & Research methods; 2 - Cell structure & Function; 3 - Chromatin; 4 - DNA replication; 5 - Gene expression; 6 - Translation & Protein routing; 7 - Signal transduction & Cell cycle; 8 - Epithelia & Glands; 9 - Connective tissue & Cartilage; 10 - Bone; 11 - Muscle; 12 - Nervous tissue; 13 - Histology Quiz. Using a cross-over study design each student encountered twelve conventional CAFAs and one single module in which CBL was applied; either CAFA 3 – Chromatin (on DNA build-up and chromatin structure) or CAFA 4 – Replication (on DNA replication and damage repair). The full course ended in the fourth week with a final examination that consisted of 88 multiple choice questions, of which five and six separate questions addressed the content of these two modules, CAFA 3 – Chromatin and CAFA 4 – Replication, respectively.

At the start of the course, all 462 students received information outlining the current study, and written consent (that covered approval for analysis of student-instructor interactions, on-line surveys and summative exam results) was obtained from 392 students prior to participation. The day after completion of module CAFA 4 – Replication, the volunteering students received an invitation by email to participate in an online survey on their CBL experience. At the end, 3 students that provided written consent did not partake in the final exam, leaving 389 participants to be included in the study. All relevant data collected was used anonymized, with prior ethical approval from relevant national and local review committees.

### Software package

All CAFA modules were built using the Lectora Online (Trivantis Corporation Inc.) authoring tool. To enable i) incorporation of certainty-based marking of question scores, ii) display of current and cumulative scores on each module page, and iii) full reporting on per-question and overall performance on a Results page at the end of the module, a customized CBL add-on (The Courseware Company BV, Utrecht, The Netherlands) was applied. For marking we adhered to the standard scoring matrix (+ 3; + 2; + 1; 0; − 2 and − 6 points) used by Gardner-Medwin & Curtin [24] and added a green to red background color scale to highlight the scores on screen (Additional file 1). In addition, total scores reflecting the number of correct and incorrect answers and the corresponding cumulative certainty-based mark were displayed on each question page. The CBL-based CAFAs ended with a final “Results” page displaying a summary of obtained results with generalized feedback and providing an option to inspect, store and print the certainty-based scores per individual questions (Additional file 2). Examples of the CBL add-on coding steps are illustrated in Additional file 3. All CAFAs were published as html packages and uploaded in the appropriate course map in the university’s digital learning environment (Blackboard Inc.), and adaptive release options were applied to direct the student cohorts to the appropriate standard or CBL-based CAFA modules.

### Study design

The study population consisted of 462 students (296 females, 166 males, age 17 to 19 years) reading Medicine or Biomedicine at Radboud University in the 2014–2015 academic year. The Cell Biology course, that set the stage for our study, runs in the third quarter of the first year, and is the sixth course the students take. Using a random number generator, the student administration office assigned the medical and biomedical students to 22 and 8 teams of 15–16 students each, respectively. For CAFAs, these student teams were merged to four fixed groups (of seven or eight teams) to fit the available computer rooms. The course schedule stipulated when each group would use the computer practical room to take a particular CAFA with instructor assistance available. Experienced instructors were present during each CAFA. For CAFA 3 – Chromatin and CAFA 4 – Replication the instructors kept record of the number and duration of any student inquiry during the session. For CAFA 3 - Chromatin (consisting of 30 question pages) two student groups took the conventional module, whereas two other groups took the CBL-based version. The next day, the latter two groups took the conventional version of CAFA 4 – Replication (comprising 37 question pages), and the other groups took the CBL-based variant. CAFAs remained accessible throughout the rest of the course, for student self-assessment outside course hours, and selective admission to the appropriate module was maintained via adaptive release in Blackboard. This cross-over design (Additional file 4) balanced CBL effects for all students on final exam results and also generated two independent measurements of CBL effects on final exam results (using chromatin- and replication-related exam questions separately).

### Exam score analyses

The examination at the end of the four-week Cell Biology course consisted of 88 multiple choice questions. Answer sheets were optically scanned and analyzed automatically, including a correction for guessing. Briefly, questions answered correctly attributed one full point to the candidate, unanswered questions delivered no points, and incorrect answers resulted in penalty points subtracted. The number of points subtracted depends on the number of alternative answers in the pertinent question; 2-choice questions resulted in − 1 point when answered incorrectly, and for questions with 3, 4 or 5 alternatives this was − 0.5, − 0.33 and − 0.25 points, respectively. Final scores for the questions on the subjects covered by CAFA 3 – Chromatin (questions 12–16) and CAFA 4 – Replication (questions 17–22), respectively, were compared between CBL and control groups, using the scores for exam questions 1–11 and 23–88 that were not related to the topics “chromatin” and “replication”, to benchmark both cohorts.

### Statistical analysis

Student-instructor interaction times were analyzed with Wilcoxon’s rank sum test using the statistics software R version 3.5.1 [25]. Item analysis of the final examination was performed in Microsoft Excel’s spreadsheet environment. Scores of the individual multiple-choice questions were binary transformed to values of 1 and 0 for correct and incorrect answers, respectively. Item difficulty, *p*′, was calculated as the fraction of students who answered correctly to that item with a correction for random guessing:

$$ {p}^{\hbox{'}}=p-\left(\frac{1-p}{k-1}\right). $$

Here, *p* is the fraction of students who answered correctly, *k* is the number of alternatives in the item. Item discrimination was calculated as *R*_*ir*_, the point-biserial “item – rest” correlation coefficient between an item’s score and the examination’s score after removing the item score from the total examination score [26]. The internal consistency of the examination was estimated using Cronbach’s alpha coefficient [26, 27].

## Results

### Participation

**Fig. 1:**
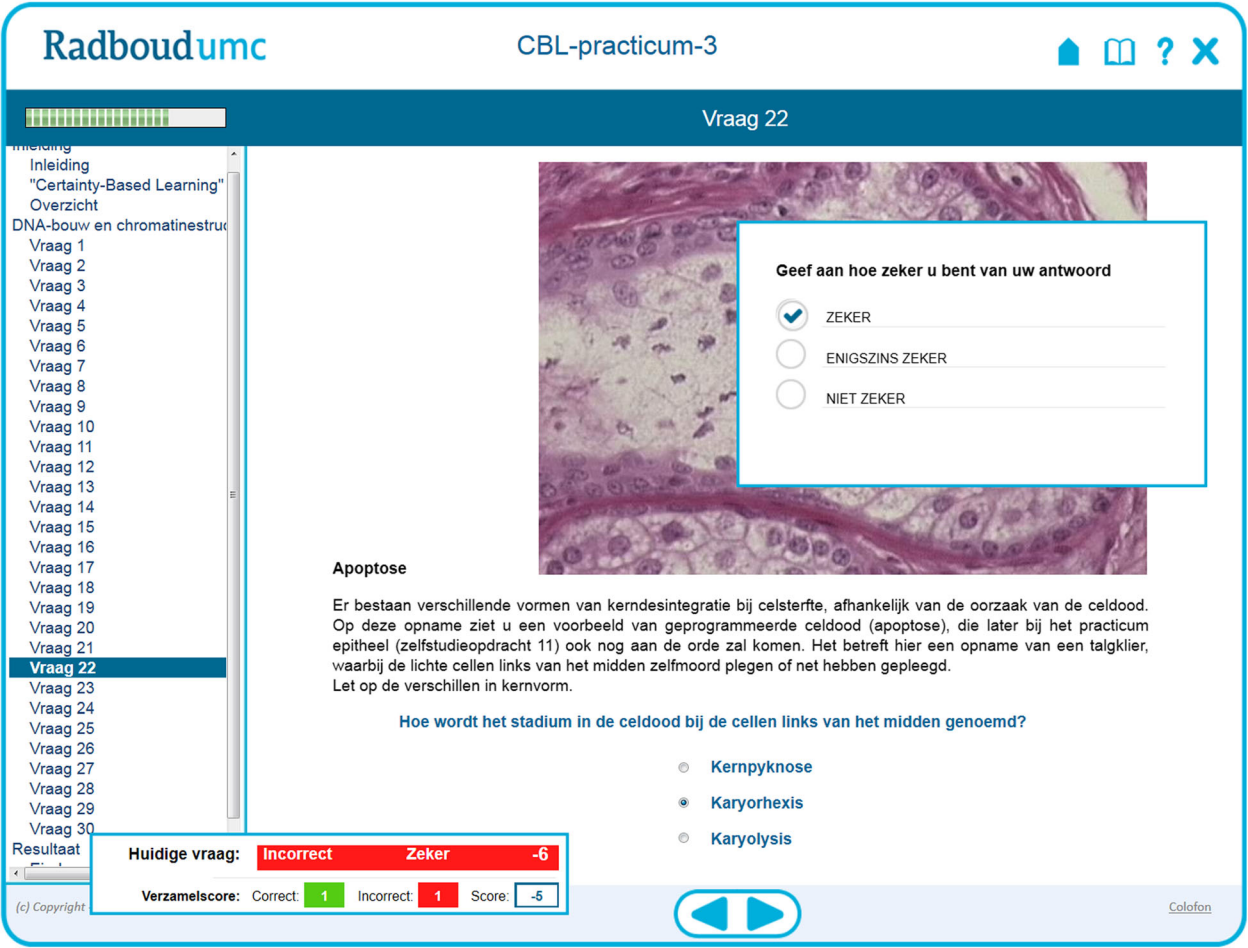
CBL display in our computer-assisted formative assessment modules. A representative page (in Dutch) from module CAFA 3 – Chromatin that includes certainty-based learning is shown. Only after answering the knowledge question (bottom part, background field) the certainty question (box in the right upper part) appears, and following its completion the scoring box (lower left) appears. The certainty question translates as “indicate how confident you are about your answer: sure (ticked in the example), partly sure, uncertain”. The scoring box displays in the top row the scoring on the current question (in Dutch: “Huidige vraag”) on the knowledge (here “incorrect”) and the certainty (here “sure” or “zeker”) answers, followed by the combined mark (in this case − 6) displayed on the corresponding background (see Additional file 1). The bottom row in the scoring box displays the cumulative score (in Dutch: “Verzamelscore”) that includes the total number of correctly and incorrectly answered knowledge questions (on a green and red background, respectively) and the sum of all individual certainty-based scores (here “-5”)

**Fig. 2:**
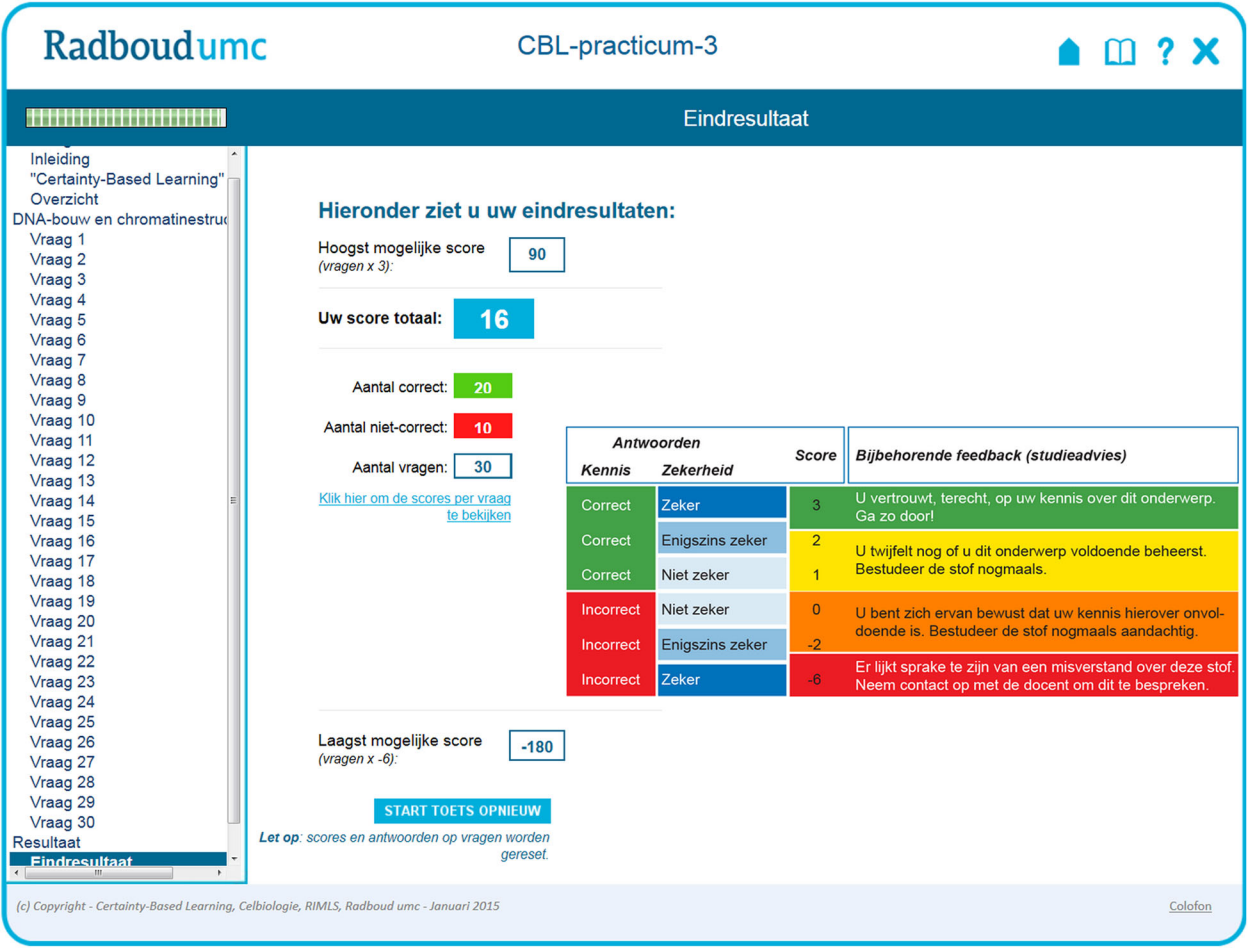
Cumulative feedback to students based on certainty-based learning as displayed in our two CAFA modules. By navigating to the results (in Dutch: “Resultaat”) page at the end of the CBL-based CAFA module, students receive integral feedback on their results. From top to bottom, on the left side in the large window the following items are displayed: the maximum score possible (the number of questions times 3), the total score by the student (on a blue background), the number of correct answers, the number of incorrect answers, the total number of questions (30), a link to inspect (and print or download) the scores per question (see Additional file 2), the minimum score possible (the number of questions times − 6), and finally a button (“START TOETS OPNIEUW”) to reset all variables and restart the formative test. The table on the right is providing feedback based on the certainty-based marks. Additional file 1 contains an English version of this table

To test whether certainty-based learning would provide an added value to computer-assisted formative assessments we introduced CBL questions in two consecutive -- and from a topic and size point of view comparable -- CAFA modules in our first-year cell biology course for (bio)medical students (Fig. 1). In addition to the scoring matrix [24] we included background color coding to highlight the combined knowledge and certainty scores (Additional file 1). Students could also inspect a results summary with generalized feedback (Fig. 2) and store and print certainty-based markings per question (Additional file 2).

During the opening lecture of the course students were informed about our study on the application of CBL in CAFAs. During the first CAFA module students received an information leaflet explaining background, purpose and design of the study as well as a written consent form. The participation rate was 85% (392 out of 462). During the modules CAFA 3 – Chromatin and CAFA 4 – Replication that were central in this study the number of participants was comparable (Additional file 4). Shortly after their participation in CAFA 4 – Replication, students were invited to complete an online survey consisting of 19 questions (Additional file 5) on their experience with the CBL-based CAFA module. The questionnaire was completed by 41% of the participants (159 students). It is of note that the vast majority of students (94%) indicated they had no prior experience with certainty-based marking.

### Student-instructor interactions

**Fig. 3:**
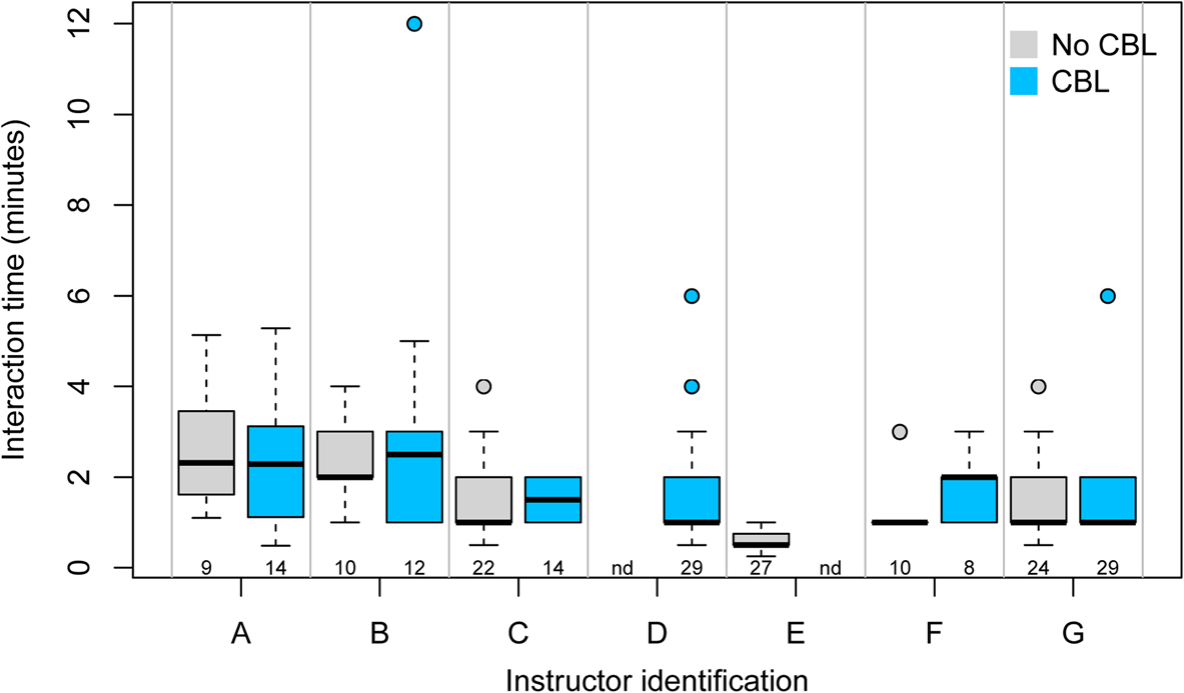
Duration but not frequency of student-instructor interactions is increased upon inclusion of CBL. Student-instructor interactions were logged during supervised CAFA sessions for modules CAFA 3 – Chromatin and CAFA 4 – Replication. Four sessions were run per module, two of which exploited CBL (light blue boxes), and in total seven different instructors (A-G) were involved. One instructor supervised only CBL-based sessions (D) and another only CAFAs that lacked CBL (E). The number of interactions per instructor for each CAFA type is listed below the box plots. Horizontal black bars indicate median interaction time (in minutes), boxes indicate the interquartile range (IQR), whiskers indicate 1.5 × IQR. Nd = not determined

At compulsory time slots during the course the students participated in CAFA modules, usually in couples of two and sometimes three students per computer terminal. We anticipated that teams confronted with a maximum negative score of − 6 (signaled by a red background, and with the feedback message “It looks like some misconceptions on this topic exist. Please contact the instructor and discuss the matter.”; Additional file 1) during supervised CBL-based CAFAs would be more inclined to approach available instructors to receive customized feedback, clarifications and explanations compared to peers that took the standard CAFA module. In contrast to our expectation, however, the number of clarification requests per student was not noticeably influenced by the provision of certainty-based marks (Fig. 3). During the four sessions per CAFA module, on average 0.22 consultations per student were recorded, with no significant difference between the control group or CBL-experiencing students during CAFA 3 or 4. The duration of the interactions, however, was on average 20 s longer during CBL-based CAFA sessions when compared to conventional CAFA versions (*p* < 0.01); median interaction times during CBL sessions were 110 s (range: 60–150 s over 102 interactions) and during the conventional versions this was 90 s (range: 60–140 s over 106 interactions).

### Student experiences

**Fig. 4:**
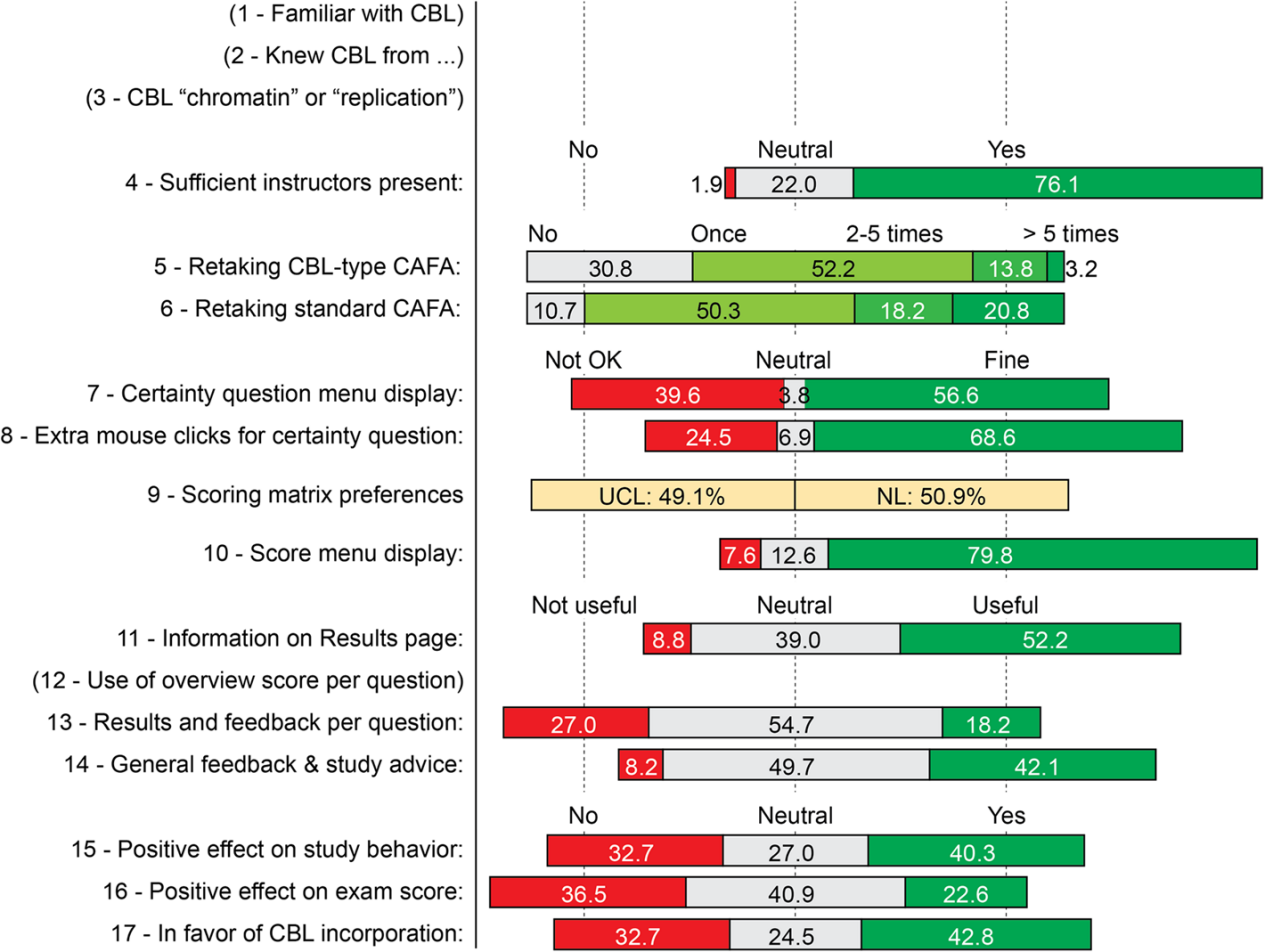
Student responses from the online anonymous perception survey. The data reflect part of the cumulative result of an 18-question online survey (*n* = 159; 41% response rate). Concise versions of the survey questions are shown on the left, distribution of choices is displayed on the right. Answers to the questions 1–3 and 12 (in parentheses) are not represented. Bar sizes are proportional to the indicated percentages and corresponding choice options are displayed above the relevant bars. Question 18 represented an open invitation for further remarks. Seventeen of the 159 students had heard of CBL before (question 1), either via contacts (5), high school experience (5) or other means (question 2). Eighty-two students worked on CBL-based CAFA 3 – Chromatin and seventy-seven on CBL-based CAFA 4 – Replication (question 3). In question 12 students were asked how often they used the possibility to save, store or print their test results as provided at the end of the CBL-CAFA (Additional file 2). Only twenty-six students (16.3%) actually used this option, and two of them did so more than once. The text of the online survey, with full description of the questions, is provided as Additional file 5

We also anticipated that CBL in CAFA modules would enhance student’s self-reflection about their knowledge levels and that this would result in targeted study activities and, ultimately, improved exam scores. To monitor whether this expectancy matched with the students’ experiences, an online questionnaire on CBL inclusion, with ample space for comments, was sent to all participants (Fig. 4). Of the 159 students that completed the survey, 82 had worked with CBL-based CAFA 3 – Chromatin and 77 with CBL-based CAFA 4 – Replication. Five reported to have previous CBL experience and another five knew the method from hear-say. A quarter of the students indicated they disliked the requirement of additional mouse-clicks to answer each CBL question, and 33% preferred not to work again with CBL-based modules during their studies. Most of these 52 students not looking forward to future CBL-based CAFAs indicated they had no problem with the additional CBL question but rather lacked confidence in the positive effects of CBL on study behavior and efficacy (33 and 36 students, respectively). Around 25% of the 159 students responded neutral but 43%, the largest group, clearly stated that they would favor CBL-inclusion in future CAFA modules (Fig. 4). The majority of students found the information as provided on the CBL Results page (Fig. 2) useful. Only 15% used the option to store or print an overview of their knowledge and certainty scores per question (Additional file 4) and just two students did so multiple times. Remarkably, when asked about the effect of the certainty-based learning on their study behavior, 40% indicated to have experienced a positive contribution to their self-assessment but only 23% expected a positive effect on the exam score (Fig. 4).

### Summative examination scores

**Fig. 5:**
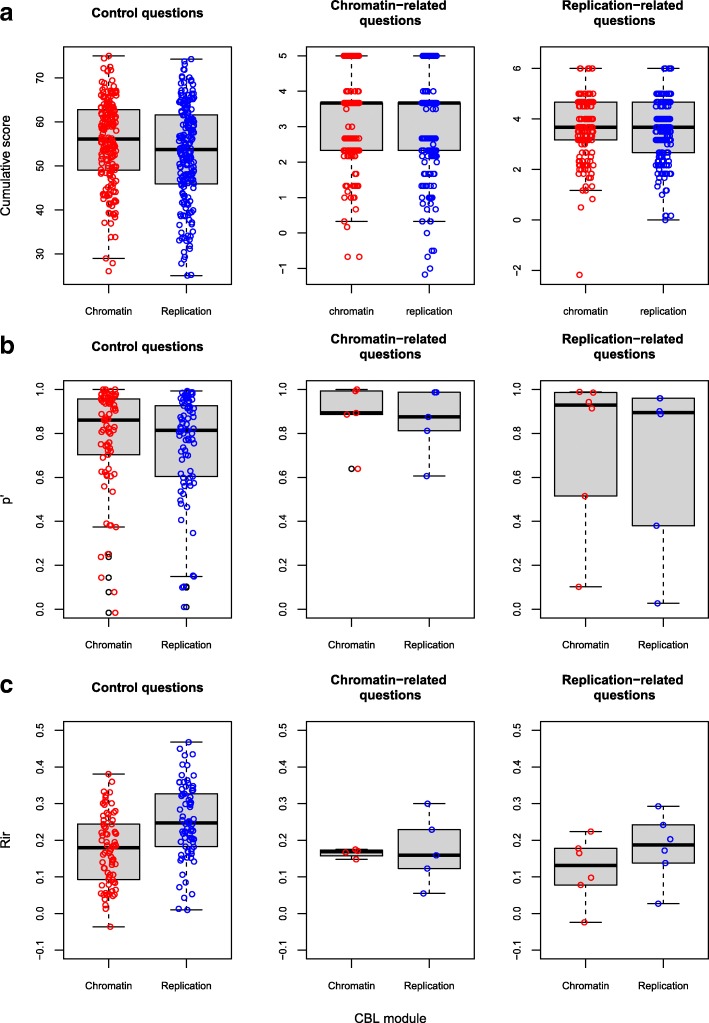
Item analysis of exam scores provides no evidence for an effect of CBL on summative assessment performance. **a** Cumulative exam scores corrected for guessing of students taking CBL-based CAFA 3 – Chromatin (red symbols) or CBL-based CAFA 4 – Replication (blue symbols). Control questions are 88 exam questions not specifically related to the topics chromatin and replication, whereas the 5 chromatin- and 6 replication-related questions were designed to specifically interrogate these respective topics. **b** p’-values for control questions, chromatin-related questions and replication-related questions. **c** Item-rest correlations of the three question sets. In all panels the horizontal black bars indicate median values, boxes indicate the interquartile range (IQR), and whiskers indicate 1.5 × IQR

The course, involving nine additional conventional CAFA modules, ended around two weeks after the CBL experiences, with an exam consisting of 88 multiple choice questions. Two small subsets, of 5 and 6 questions each, addressed the topics dealt with in CAFA 3 – Chromatin and CAFA 4 – Replication, respectively. In total 389 students who filled out and signed the informed consent document actually participated in the exam; 187 had worked with the CBL version of CAFA 3 (group “Chromatin”) and the other 202 experienced CBL during CAFA 4 (group “Replication”). Performance on questions unrelated to the topics covered in these two CAFAs was used to benchmark the two groups (Fig. 5). The average score for group “Replication” on the 77 control questions was slightly lower than that of group “Chromatin” (52.5 ± 11.8 versus 55.5 ± 9.7 points; *p* < 0.01). A comparison of scores for both groups on the chromatin- and replication-related questions revealed no statistically significant differences between the groups (Fig. 5a).

We analyzed test results for each individual question using conventional psychometric indicators. The reliability of the 88-question exam, as represented in Cronbach’s alpha scores, was 0.77 for group “Chromatin” and 0.87 for group “Replication” (data not shown). Scores higher than 0.70 indicate a good reliability and internal consistency of the test. Due to the ethical consideration for using a crossover study design we cannot compare data to a student cohort that did not experience CBL at all during the CAFA modules. Also, Cronbach’s alpha scores for the five CAFA 3 (chromatin) and six CAFA 4 (replication) related questions in separation cannot reliably be determined because of the small number of questions.

When analyzing the p’-values for each question, again the control set questions (1–11 and 23–88) suggested a small underperformance of the group “Replication” (Fig. 5b). For the number of questions addressing CAFA 3 – Chromatin (questions 12–16) or CAFA 4 – Replication (questions 17–22) subjects, the corresponding p’-values are well comparable between both cohorts. Also, the correlation between an individual question score and the remaining exam question scores (item-rest correlation, *R*_*ir*_) does not reveal effects of CBL inclusion (Fig. 5c).

## Discussion

During the last two decades the use of certainty-based learning, usually in combination with computer-based multiple choice-questioning, has been explored and its effectiveness been studied in different contexts. Following the pioneering work by Gardner-Medwin and colleagues [11–15, 24, 28, 29], the method has been exploited to support diverse educational goals [18, 19, 21–23, 30]. Over the years, evidence has accumulated that the incorporation of CBL during formative tests is well-appreciated [19, 21], feeds the students’ confidence in their knowledge [22], and supports the learning process [19, 21]. No evidence for an effect of high or low risk-taking personalities on CBL-based exam scores has been encountered thus far and in fact CBL may help in raising student awareness about under- or overconfidence [29]. Its use in consecutive tests, where incorrectly answered questions are fed into follow-up tests, effectively invites students to work on their knowledge gaps [20]. Furthermore, CBL provided important indications how to reshape tests and educational courses [22, 23]. Studies mostly analyzed pre/post-module effects on (formative) assessments by individuals that all experienced CBL in the respective course. In the current study we tested whether a computer-assisted formative assessment that exploits CBL provides added value with respect to learning behavior and (summative) exam scores. The use of a cross-over study design on a large cohort of (CBL-naïve) students enabled us to probe, two times in a row, whether groups confronted in the course with CBL during a formative assessment on a certain topic would outperform their peers on exam questions dealing with that topic at the end of the course.

All except a few students involved in the study were novice users of CBL. Irrespective of this, many students appreciated CBL, and a considerable percentage expected beneficial effects on exam scores. We expected to observe that CBL inclusion in CAFAs would improve study behavior, having students more actively seeking feedback from instructors and producing better scores during subsequent exams. We found, however, that students who had worked with a CBL-based CAFA only differed from their peers in engaging in interactions with available staff that took around 20% longer. Students mostly performed the CAFAs in pairs, and perhaps ad hoc discussions with neighbors provided peer feedback that may have acted as a surrogate certainty-based test, making it more difficult to detect measurable effects of CBL on student performance. The sensitivity of our study may also be hampered by the fact that only two out of the thirteen formative assessments were transformed into CBL-based CAFAs and, consequently, that just a small subset of questions out of the 88-question final exam would be informative in this respect. On the other hand, the current set-up allowed the remaining exam questions - that cover subjects related to the other, conventional CAFAs - to serve as a calibration tool for cohort differences. Furthermore, the cross-over design eliminated the ethical issue of withholding a potentially powerful study aid from half of the students and a consequent bias in final course grades. A potential downside may be that for group “Chromatin” any effect of their CBL-based experience might influence their subsequent study behavior, also while taking CAFA 4 – Replication, and thus confound detection of any effects.

The majority of students in the study were familiar with our CAFA E-tools but encountered CBL for the first time. Our curriculum mainly consists of courses taught with conventional teaching methods, and it can well be envisaged that the efficiency and efficacy of a novel E-tool such as our CBL-implementation is perceived as an isolated event, not embedded in the curriculum. This may negatively affect the results of E-tool usage. Such factors could explain why we did not observe the effects we anticipated. It emphasizes that the implementation of a new tool should be guided carefully and be allowed to evolve over subsequent course iterations [31, 32].

Addition of more CAFAs based on CBL in our course might have increased the sensitivity of our study. This, however, requires a convenient authoring tool to incorporate CBL in existing CAFA modules. The script we used to convert two existing CAFAs into CBL-compliant versions is quite laborious (Additional file 3) as currently full support for CBL is realized only in the LAPT-lite software [16]. As a consequence, multiple certainty-based learning adepts (e.g. [24]) urge for convenient implementation of CBL functionality in e-learning authoring tools such as Blackboard, BrightSpace, Lectora Online, Moodle, Questionmark or WebCT. Customer-friendly CBL embedding in e-learning tools would greatly facilitate research towards its educational applicability. It is of note that such applications are not limited to students’ study performance and outcome; CBL inclusion in formative assessments may impinge on test designs and course evaluations, hence represent an important and useful assessment tool for teaching staff as well.

## Conclusion

Inclusion of certainty-based learning in computer-assisted formative assessments is well appreciated by students and staff. Our study revealed no overt effects on student-instructor interactions or student study performance. Development of a user-friendly CBL option in e-learning authoring tools will enable more studies towards its added value in (bio)medical educational programs.

## Additional files

Additional file 1: Feedback to students based on certainty-based learning as displayed in Cell Biology computer-assisted formative assessment modules. Both the Dutch version used in the current study (A) and the English equivalent (B) are shown. Scores corresponds to the scheme used at the University College London [24]. An incorrect answer will not lead to a penalty if students indicate to be uncertain (score 0 for “not sure”). Wrong answers that are provided with confidence, however, result in a firm warning through negative points (score = − 6). Students that answer correctly but are not sure will not be able to gain maximal scores (1 or 2 instead of 3 points). Only students that provide a correct answer AND are fully confident about this will get the full bonus; the student knows he/she knows. At the end of a CAFA module one can then provide students with tailored feedback, including study advice, specified for each CBL score category. (PNG 335 kb)

Additional file 2: CBL results for individual questions are available for on-screen inspection, printing and filing. Upon selecting the link “Klik hier om de scores per vraag te bekijken” on the final results page (Fig. 2) in CBL-based CAFA modules, a full list of results for individual filled-out questions is displayed on the background color assigned to the respective certainty-based learning score (see Additional file 1). The various columns present the question number (VRAAG), the knowledge score (STATUS), the certainty level (ZEKERHEID) and the certainty-based SCORE. Note that only questions that were fully answered will be shown (in this case all 30). (PNG 208 kb)

Additional file 3: CBL script in Lectora Online. A) Example set-up of a program with 14 questions. B) Detailed look at CBL-module in question 4. C) Upon entering the page “results”, student’s performance is calculated and shown. D) Additional actions required to display student results per question. (PNG 1435 kb)

Additional file 4: Schematic overview of the applied cross-over study to investigate a possible added value of certainty-based learning in computer-assisted formative assessment modules. A description of the various components and steps in the study is provided in the Methods section. (PNG 74 kb)

Additional file 5: Questionnaire with 19 questions, including a request for further comments, on CBL use (in Dutch). (PDF 75 kb)

## Abbreviations

CAFA: Computer-assisted formative assessment
CBL: Certainty-based learning
