# Supplementary figures and images for: Certainty-based marking in a formative assessment improves student course appreciation but not summative examination scores
Source: BMC Med Educ. 2019 May 31;19:178. doi: 10.1186/s12909-019-1610-2 (PMC6544949; doi:10.1186/s12909-019-1610-2)

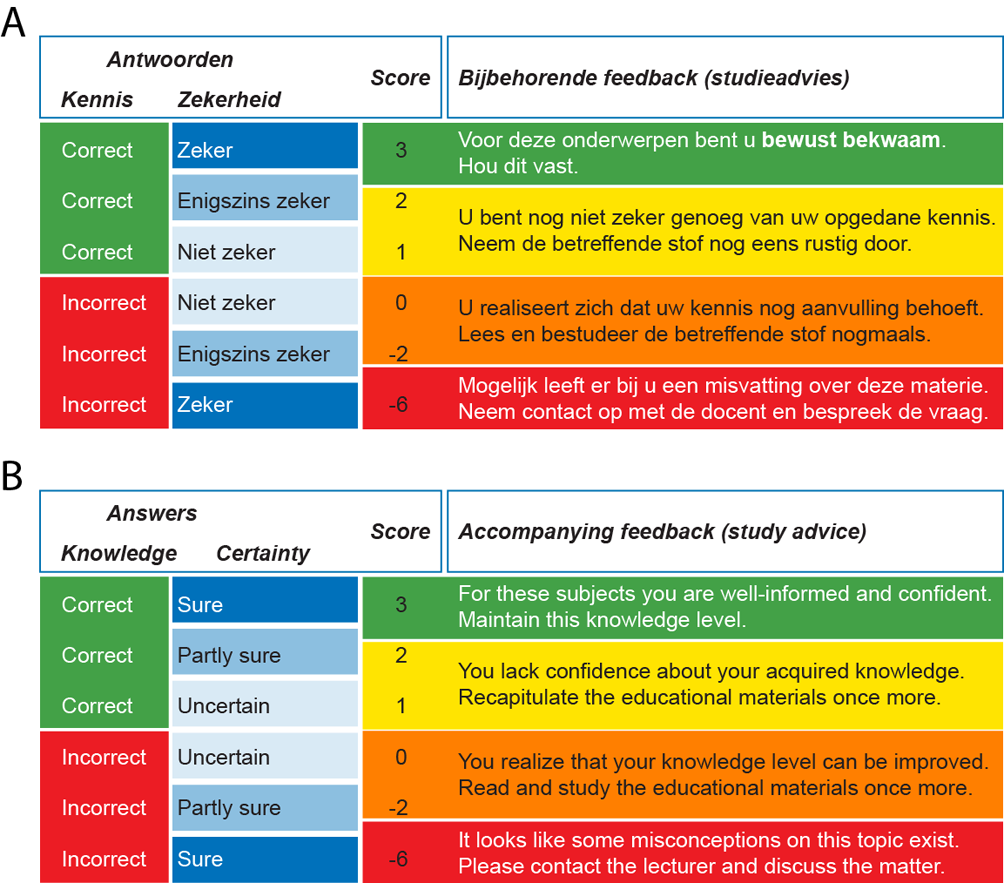

Supplement: Supplementary file 1 — Feedback to students based on certainty-based learning as displayed in Cell Biology computer-assisted formative assessment modules. Both the Dutch version used in the current study (A) and the English equivalent (B) are shown. Scores corresponds to the scheme used at the University College London [24]. An incorrect answer will not lead to a penalty if students indicate to be uncertain (score 0 for “not sure”). Wrong answers that are provided with confidence, however, result in a firm warning through negative points (score = − 6). Students that answer correctly but are not sure will not be able to gain maximal scores (1 or 2 instead of 3 points). Only students that provide a correct answer AND are fully confident about this will get the full bonus; the student knows he/she knows. At the end of a CAFA module one can then provide students with tailored feedback, including study advice, specified for each CBL score category. (PNG 335 kb) [file 12909_2019_1610_MOESM1_ESM.png]

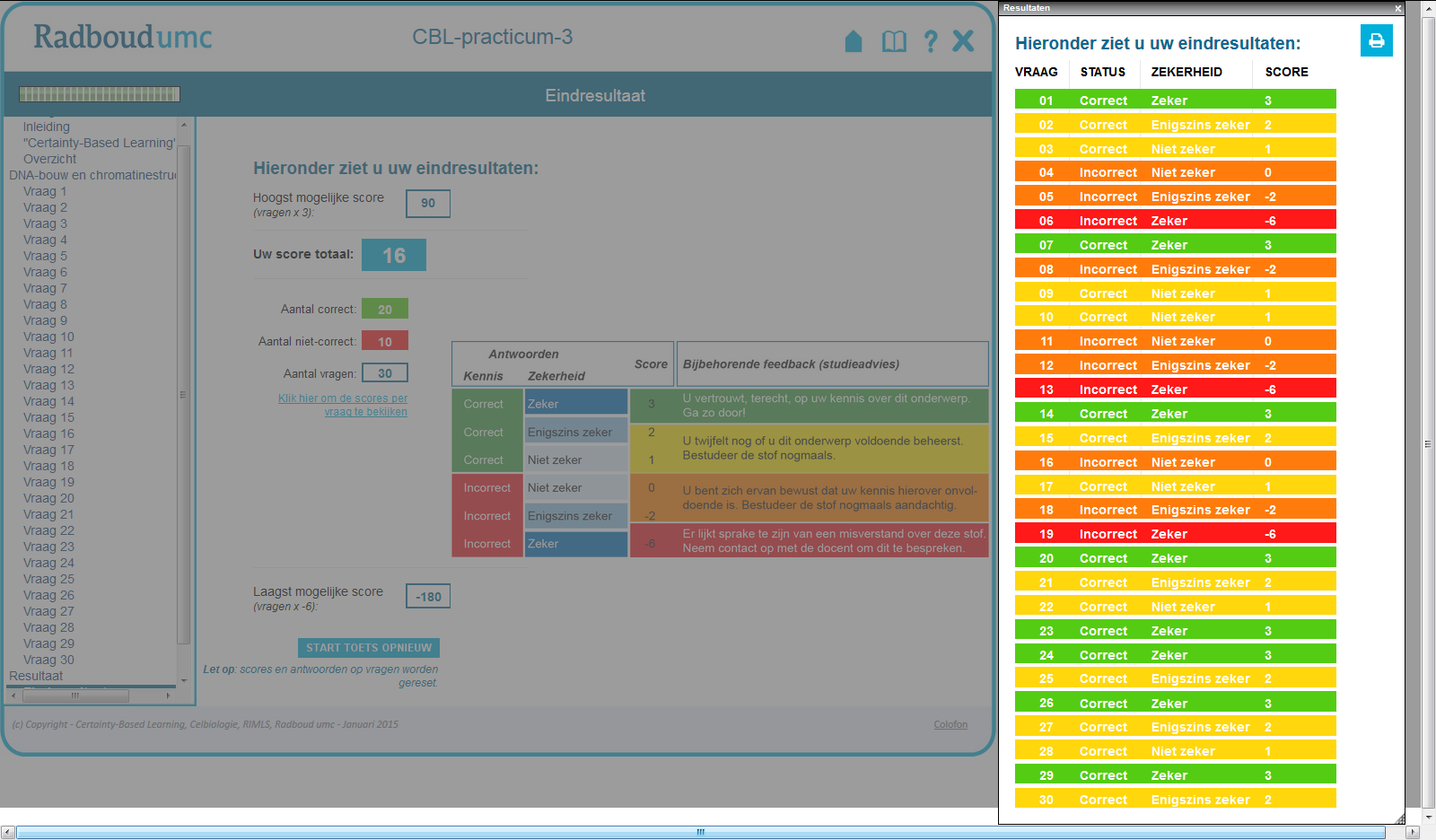

Supplement: Supplementary file 2 — CBL results for individual questions are available for on-screen inspection, printing and filing. Upon selecting the link “Klik hier om de scores per vraag te bekijken” on the final results page (Fig. 2) in CBL-based CAFA modules, a full list of results for individual filled-out questions is displayed on the background color assigned to the respective certainty-based learning score (see Additional file 1). The various columns present the question number (VRAAG), the knowledge score (STATUS), the certainty level (ZEKERHEID) and the certainty-based SCORE. Note that only questions that were fully answered will be shown (in this case all 30). (PNG 208 kb) [file 12909_2019_1610_MOESM2_ESM.png]

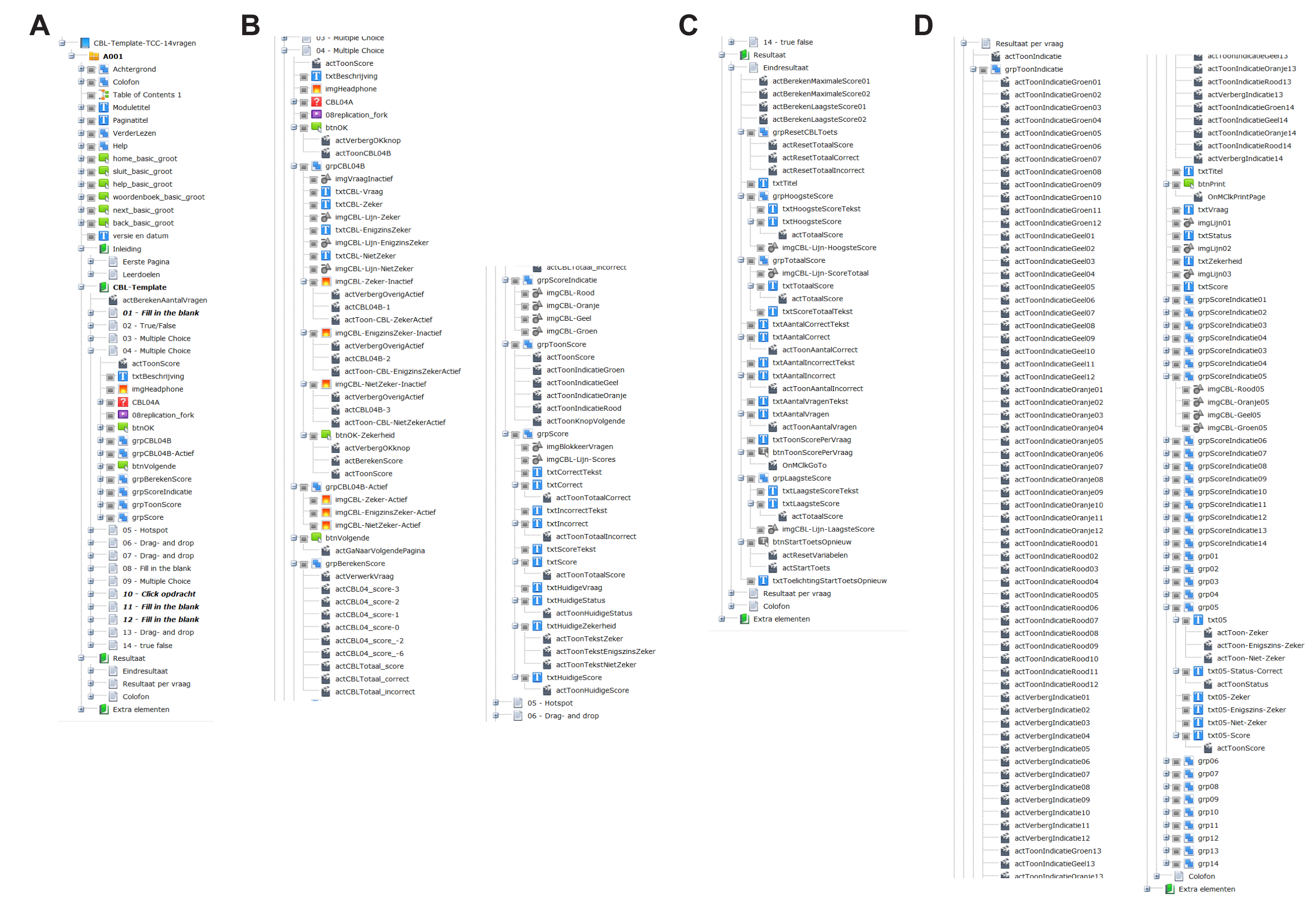

Supplement: Supplementary file 3 — CBL script in Lectora Online. A) Example set-up of a program with 14 questions. B) Detailed look at CBL-module in question 4. C) Upon entering the page “results”, student’s performance is calculated and shown. D) Additional actions required to display student results per question. (PNG 1435 kb) [file 12909_2019_1610_MOESM3_ESM.png]

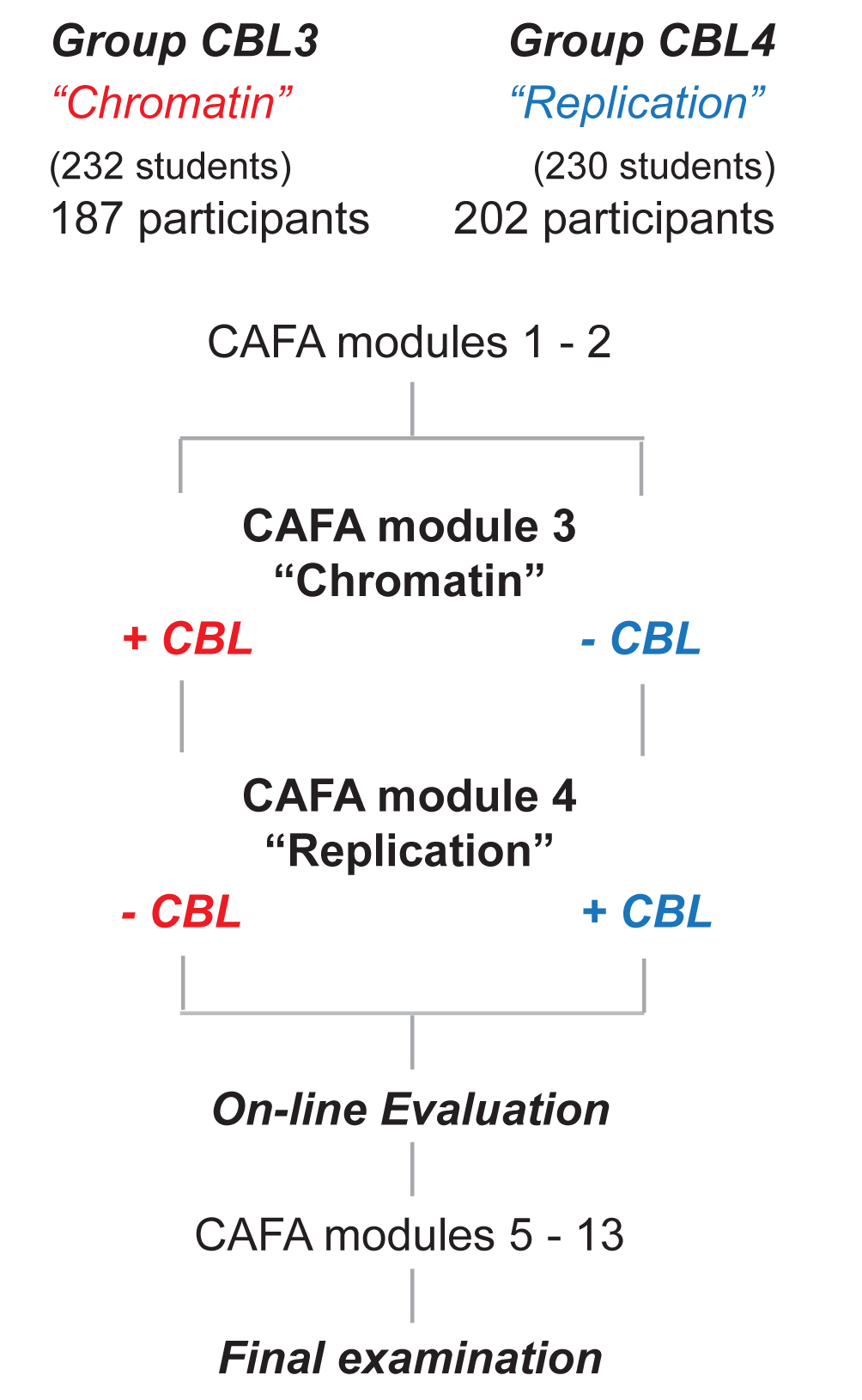

Supplement: Supplementary file 4 — Schematic overview of the applied cross-over study to investigate a possible added value of certainty-based learning in computer-assisted formative assessment modules. A description of the various components and steps in the study is provided in the Methods section. (PNG 74 kb) [file 12909_2019_1610_MOESM4_ESM.png]
