# Supplementary material for: Certainty-based marking in a formative assessment improves student course appreciation but not summative examination scores
Source: BMC Med Educ. 2019 May 31;19:178. doi: 10.1186/s12909-019-1610-2 (PMC6544949; doi:10.1186/s12909-019-1610-2)
Supplement: Supplementary file 5 — Questionnaire with 19 questions, including a request for further comments, on CBL use (in Dutch). (PDF 75 kb) [file 12909_2019_1610_MOESM5_ESM.pdf]

# Certainty-Based Learning

Start

www.thesistools.com

# Certainty-Based Learning

1.

**Was u voor dit blok op de hoogte van het bestaan van Certainty-Based Learning (CBL)?***

- Ja
- Nee

2.

**Waar kende u het CBL-leer principe van?***

- de middelbare school
- de basisschool
- het internet
- de krant
- leeftijdsgenoten
- ouders/oudere bekenden
- andere bronnen
- ik kende het CBL-leer principe niet

Created by PDFmyURL. Remove this footer and set your own layout? Get **a license!**

PDFmyURL

3.

**Bij welk onderdeel heeft u de CBL practicum-module gebruikt?***

- Practicum 3 (DNA-bouw en chromatinestructuur)
- Practicum 4 (DNA-replicatie en schadeherstel)

4.

**Was er voldoende assistentie aanwezig tijdens de CBL practicum-module (Practicum 3 of 4)?**

- Ja
- Neutraal/geen mening
- Nee

5.

**Hoe vaak denkt u de CBL practicum-module (Practicum 3 en 4) nog te gaan hergebruiken?***

- 0x
- 1x
- 2-5x
- >5x

6.

**Hoe vaak denkt u de andere (normale, niet-CBL) practicum-module te gebruiken?***

- 0x
- 1x
- 2-5x
- >5x

Created by PDFmyURL. Remove this footer and set your own layout? Get **a license!**

PDFmyURL

7.

**De 'zekerheidsvraag' werd over het venster met de kennisvraag geprojecteerd. Vindt u dit een probleem?***

- Ja, dit is vervelend
- Ja, maar de voordelen van CBL compenseren dit
- Nee, dit is geen probleem

8.

**Voor het beantwoorden van elke 'zekerheidsvraag' waren er extra muis-klikken nodig. Vindt u dit een probleem?***

- Ja, dit is vervelend
- Ja, maar de voordelen van CBL compenseren dit
- Nee, dit is geen probleem

9.

**De 'zekerheids-score' verliep volgens de ''University College Londen'' schaal (van 'correct&zeker =3' tot en met 'incorrect&zeker = -6'). De score zou ook geconformeerd kunnen worden aan ons Nederlandse 10-punten-systeem (van 'correct&zeker =9' tot en met 'incorrect&zeker = 0'). Wat heeft uw voorkeur?***

- University Collage Londen (UCL) -schaal
- Nederlandse (NL) -schaal

10.

**De 'score' werd linksonder op elke pagina geprojecteerd. Vond u dit een probleem?***

- Ja, het verborg delen van de inhoudsopgave en de pagina /vraag
- Neutraal/geen mening

Created by PDFmyURL. Remove this footer and set your own layout? Get **a license!**

○ Nee, het stond niet in de weg

11.

**Op de eindresultaat-pagina kon u uw 'totaalscore' vergelijken met de maximale en minimale scoremogelijkheden. Vond u dat nuttig?***

○ Nuttig
○ Neutraal/geen mening
○ Niet nuttig

12.

**Via de eindresultaat-pagina kon u alle scores per vraag oproepen en opslaan/uitprinten. Hoe vaak heeft u daar gebruik van gemaakt?***

○ 0x
○ 1x
○ 2-5x
○ >5x

13.

**Via de eindresultaat-pagina kon u alle scores per vraag oproepen en opslaan/uitprinten. Vond u dit nuttig***

○ Nuttig
○ Neutraal/geen mening
○ Niet nuttig

14.

**Aan het begin en aan het eind van de CBL practicum-module werd een tabel getoond die per score-categorie**

Created by PDFmyURL. Remove this footer and set your own layout? Get **a license!**
PDFmyURL

**feedback/studieadvies gaf. Vond u die nuttig?***

- Nuttig
- Neutraal/geen mening
- Niet nuttig

15.

**Verwacht u dat de CBL- toevoeging aan het computerpracticum een positieve uitwerking op uw STUDEERGEDRAG zal hebben?***

- Ja
- Geen mening/neutraal
- Nee

16.

**Verwacht u dat de CBL- toevoeging aan het computerpracticum een positief effect op uw TENTAMENRESULTAAT zal hebben?***

- Ja
- Geen idee
- Nee

17.

**De hamvraag, tenslotte: vindt u dat er meer computerpractica voorzien moeten worden met deze CBL-optie?***

- Ja
- Geen mening/neutraal
- Nee

Created by PDFmyURL. Remove this footer and set your own layout? Get **a license!**

18.

**Op welke manier zou de CBL module verbeterd kunnen worden?**

19.

**Verdere opmerkingen/suggesties/feedback/commentaar**

verstuur enquête



Bedankt voor het invullen van deze enquête

*www.thesistools.com*

Created by PDFmyURL. Remove this footer and set your own layout? Get **a license!**
